# Viral metagenomics demonstrates that domestic pigs are a potential reservoir for Ndumu virus

**DOI:** 10.1186/1743-422X-9-218

**Published:** 2012-09-24

**Authors:** Charles Masembe, George Michuki, Maria Onyango, Cecilia Rumberia, Martin Norling, Richard P Bishop, Appolinaire Djikeng, Stephen J Kemp, Alan Orth, Robert A Skilton, Karl Ståhl, Anne Fischer

**Affiliations:** 1 Department of Biological Sciences, Makerere University, Kampala, Uganda; 2 International Livestock Research Institute (ILRI), Nairobi, Kenya; 3 Biosciences Eastern and Central Africa (BecA) –ILRI Hub Nairobi, Nairobi, Kenya; 4 Swedish University of Agricultural Sciences (SLU), Uppsala, Sweden; 5 International Centre of Insect Physiology and Ecology, icipe, Nairobi, Kenya

**Keywords:** Metagenomics, Ndumu virus, Pigs, Reservoir, Zoonoses

## Abstract

**Background:**

The rising demand for pork has resulted in a massive expansion of pig production in Uganda. This has resulted in increased contact between humans and pigs. Pigs can act as reservoirs for emerging infectious diseases. Therefore identification of potential zoonotic pathogens is important for public health surveillance. In this study, during a routine general surveillance for African swine fever, domestic pigs from Uganda were screened for the presence of RNA and DNA viruses using a high-throughput pyrosequencing method.

**Findings:**

Serum samples from 16 domestic pigs were collected from five regions in Uganda and pooled accordingly. Genomic DNA and RNA were extracted and sequenced on the 454 GS-FLX platform. Among the sequences assigned to a taxon, 53% mapped to the domestic pig (*Sus scrofa*). African swine fever virus, Torque teno viruses (TTVs), and porcine endogenous retroviruses were identified. Interestingly, two pools (B and C) of RNA origin had sequences that showed 98% sequence identity to Ndumu virus (NDUV). None of the reads had identity to the class Insecta indicating that these sequences were unlikely to result from contamination with mosquito nucleic acids.

**Conclusions:**

This is the first report of the domestic pig as a vertebrate host for Ndumu virus. NDUV had been previously isolated only from culicine mosquitoes. NDUV therefore represents a potential zoonotic pathogen, particularly given the increasing risk of human-livestock-mosquito contact.

## Background

**Figure 1:**
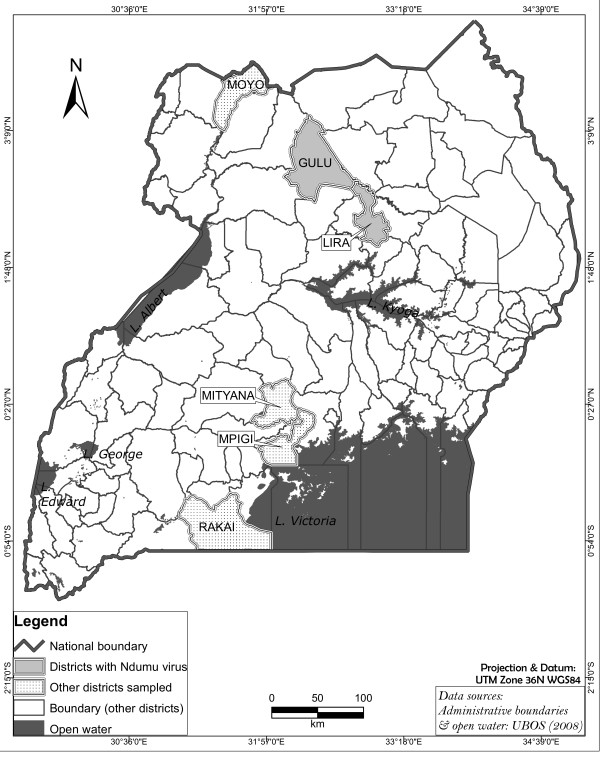
**Map of Uganda. **In grey, districts where samples were collected, in dark grey, districts where NDUV was identified.

The rising demand for livestock products in Africa has resulted in an increased use of intensive pig production systems across the continent. Pigs are frequently preferred to other livestock species due to their relatively rapid growth rate and large litter sizes. In Uganda, the pig production industry is rapidly developing, as it has the potential to provide financial returns over a relatively short time [1]. However, intensification of pig production leads to increased contact between humans and domestic animals with possible public health consequences. Zoonoses such as Cysticercosis, Swine Influenza virus, Nipah virus, Menangle virus, porcine Hepatitis E, *Staphylococcus aureus* and *Streptococcus suis* have been reported in pigs and there is clear potential for domestic swine to act as a reservoir for many emerging and re-emerging infectious diseases [2-7]. A complete picture of other potential zoonoses carried by domestic pigs that could potentially cross over to humans due to close confinement of pigs and humans, especially in smallholder pig farmers in Africa, is still lacking. Early identification of the pathogen spectrum and diagnosis of potential zoonoses in domestic animals in close contact with humans is therefore important for public health surveillance and development of early warning systems. Due to resource constraints, such surveillance is currently minimally implemented in developing countries, such as Uganda. Recent advances in next generation, high-throughput sequencing technologies offer rapid ways to identify and analyze the presence and diversity of emerging pathogens [8,9]. Viral metagenomics is therefore increasingly applied in veterinary epidemiology, as exemplified in the recent identification of a novel porcine boca-like virus [7] and Torque teno sus viruses 1 and 2 [10]. In this study, during a routine general surveillance for African swine fever, serum samples from 16 domestic pigs in five districts in Uganda (Figure 1) were analyzed using 454 pyrosequencing to characterize viral diversity.

## Findings

### Methodology

Serum samples from 16 domestic pigs were collected from five localities in Uganda (Figure 1). The sampling was carried out as part of a study on African swine fever (endemic in the region) from localities with suspected outbreaks of the disease. Serum samples from pigs originating from the same geographical locality were combined to generate a total of six pools from across the country (A-Moyo; B-Lira; C-Gulu; D-Mityana; E-Mpigi). The samples were filtered through a 0.22 μm filter followed by DNA and RNA extraction using a Qiamp DNA Mini kit (Qiagen) and TRIzol reagent (Invitrogen), respectively. DNA and RNA were amplified using the modified random priming mediated sequence independent single primer amplification (RP-SISPA) methodology [11]. Each amplified sample was further processed as described for shotgun library preparation in GS FLX 454 technology. The sequencing reads were trimmed to remove SISPA primers and barcodes, and only reads with a length greater than 50 bp were retained. Low complexity repeats were masked using Repeatmasker (*RepeatMasker Open-3.0*.1996-2010 http://www.repeatmasker.org) and sequences with more than 50% repeats were excluded. The sequences in each pool were assembled using the Newbler assembler version 2.5.3 with default settings (Roche*.* Genome Sequencer FLX Data Analysis Software Manual. *Mannheim, Germany: Roche Applied Science, 2007*). Contiguous sequences (contigs) and reads which did not assemble into contigs were categorized using BLASTN and BLASTX homology searches against the non-redundant nucleotide and amino acid databases from NCBI (version June 2011). Taxonomic classification of each contig/read was investigated using MEGAN 4.0 [12].

## Results

A total of 289,038 reads with an average length of 175 nucleotides was obtained. After filtering for length and repeat content, 190,706 reads remained. Seventy-seven percent of the reads were assembled into contigs. Both BLASTN and BLASTX analyses gave similar results. Subsequently, the results that follow are from the BLASTN analysis.

For 62% of all sequences, there was no significant match within GenBank as defined by the above criteria. Among the sequences assigned to a taxon, 52%, whether DNA or RNA, mapped to the domestic pig host (*Sus scrofa*). Thirty six percent of the sequences also exhibited similarity with other mammalian genomes (17% to the family *Bovidae* and 6.5% to human); these additional matches were most likely a consequence of the currently incomplete status of the porcine genome.

A small proportion (6.2%) of the sequences mapped to DNA and RNA viruses. For the DNA searches, besides African swine fever virus, which was identified in all pools, Torque teno viruses (TTVs) were identified in two pools (A and D). TTVs are ubiquitous species-specific viruses that are currently considered non-pathogenic and have been reported to infect swine with a high prevalence [10,13].

From the RNA pools analyses, three pig serum pools (A, C, & D) showed sequences with significant sequence similarity to diverse porcine endogenous retroviruses. Of particular interest are sequences that showed highly significant identity (98%) to NDUV and were present in pools B and C of pig RNA samples from Gulu and Lira districts, respectively. To validate this observation, the distribution of the sequence reads used earlier to build contigs from the two pools were determined by BLASTN analysis. RNA pool B had 6,228 sequence reads which comprised of 2% virus, 1% bacteria, 57% *Mammalia* and 40% unknown. RNA pool C had 64,583 reads which comprised of 5% virus, 1% bacteria, 51% *Mammalia* and 43% unknown (Additional file 1: Figure S1). The *Mammalia* genomes were from pig, human and *Bovidae*. Bacteria reads in both pools had identities of less than 30 bases at below 65% identity to *Eubacterium hallii* and *Xylella fastidiosa.* The bacteria sequences may therefore have occurred by chance and hence not significant explaining why they were lost on assembly of reads to contigs. No read showed a match to the class *Insecta*.

**Figure 2:**
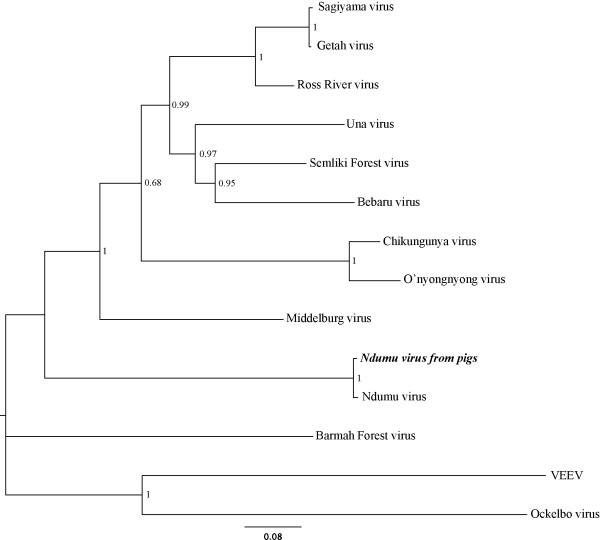
**Maximum Likelihood phylogenetic tree of selected alphaviruses protein sequences. **The NDUV from this study is in bold italics. Numbers on internal branches indicate bootstrap values for 500 replicates. VEEV: Venezuelan Equine Encephalitis Virus.

For the Ndumu virus (NDUV), a mapping assembly against the Ndumu genome sequence available in Genbank (NC_016959) using gsMapper was performed (Roche*.* Genome Sequencer FLX Data Analysis Software Manual. *Mannheim, Germany: Roche Applied Science, 2007*). Two contigs were built and the average coverage for each base was 10-fold. The mapped contigs were then masked: a minimum of two independent reads and a base quality of 20 was required for a base to be called. Masked sequences of the NDUV are available in GenBank under accession numbers JN989957-JN989958. The presence of NDUV was confirmed by amplification of 118 base pairs (bp) of the NDUV virus with NDUV-specific primers (Ndu-F1 GCCTACGTAGAACGTGCAGA and Ndu-R1 TGATGTTTCCCAACGTCACT). The PCR product was purified and sequenced on an ABI Prism 3700 DNA analyzer (Perkin-Elmer Applied Biosystems, Foster City, CA). The resulting sequences were compared to other members of the alphavirus genus from Genbank (AF069903, U73745, EF536323, AB032553, AY702913, GQ433358, X04129, AF369024, AF079456, NC_016962, NC_016959, M69205, HM147992). The protein coding regions (non-structural and structural proteins) of these sequences, and that of the NDUV sequences in this study were aligned using ClustalW [14]. In addition, a complete genome of 11688 bp of NDUV (GenBank NC_016959.1), and sequences of the Semliki Forest virus complex were added to the data set to construct phylogenetic trees. The alignment of the sequences is available as Additional file 2: Figure S2 and also as a complete file (Additional file 3: Sequence data). A neighbor-joining and maximum likelihood phylogenetic tree of the aligned protein sequences was constructed with MEGA 5.0 [15] using the Jones, Taylor and Thornton model of evolution and gamma distributed rates at sites incorporating 500 bootstrap replicates to assess the support of the phylogeny. Sequences of Venezuelan Equine Encephalitis Virus (VEEV) and Sindbis-Ocklebo virus were used as outgroups, based on previous phylogenetic studies [16]. The pig serum-derived RNA pool (B &C) sequences cluster with the currently available NDUV virus sequence as shown in Figure 2, confirming that for the first time, this virus is detected in a vertebrate host, the domestic pig.

## Discussion

In this study, a metagenomics approach was used to determine the variety of viruses in domestic pig (*Sus scrofa*) serum. In addition to the detection of mammalian sequences, it revealed the presences of some viruses for example Torque teno viruses (TTVs) and bacteria *Eubacterium hallii* and *Xylella fastidiosa* that have previously been found to occur regularly in pigs and plants, respectively. However, in the same study, a virus that has not been found before in pigs, the NDUMU (NDUV) virus, was detected. NDUV is a single stranded RNA arbovirus transmitted by mosquitoes and belonging to the *Togaviridae* family in the alphavirus genus. Very little is known about NDUV and its vertebrate hosts. It was isolated for the first time in South Africa in 1959 from *Mansonia uniformis*[17] and later in Kenya from *Aedes mcintoshi* and *A. ochraceus*[18]. Mice experimentally infected with NDUV do not survive the infection [17]. Although antibodies to the virus have been identified in humans from several African countries, no human morbidity or mortality has yet been attributed to NDUV infection [17]. However the genus Alphavirus comprises at least 24 members [19], among which are many viruses, which cause diseases in humans and other animals. Chikungunya virus is one example of an Alphavirus that was responsible for recent severe outbreaks of human disease in Eastern Africa. In humans, the symptoms associated with Alphavirus infections range from fevers and rashes, to transient or debilitating arthritis, or encephalitis [20,21]. In this study, the domestic pig has for the first time been identified as a potential vertebrate host of NDUV. NDUV therefore represents a potential zoonotic agent, given the increasing risk of human-livestock-mosquito contact as the pig industry continues to intensify, and the pig population increases in Uganda. Our discovery indicates that a focused search for the virus using reverse-transcription PCR should now be performed in human communities associated with the domestic pig populations in which we have detected NDUV.

## Competing interests

The authors do hereby declare that they have no competing interests whatsoever in this scientific work.

## Authors’ contributions

CM conducted fieldwork, contributed to field study design, contributed to lab work, draft manuscript, and final manuscript preparation. GM, MO, CR performed the laboratory experiments and contributed to the data analysis and drafting of the manuscript. RPB, AD, SJK, RS contributed to study design and manuscript preparation. MN provided python scripts. AO installed and maintained the necessary programs on the server. KS coordinated the fieldwork and contributed to field study design, and final manuscript preparation. AF performed data analysis, wrote a manuscript draft and contributed to final manuscript preparation. All authors have read and approved the final manuscript.

## Supplementary Material

Additional file 1 **Figure S1. **Taxonomic classification of sequence reads used to build contigs for RNA pools B (Gulu district) and C (Lira district) based on BLASTN (E-value <0.001) against genebank non-redundant database. Click here for file

Additional file 2 **Figure S2. **Window showing alignment of amino acids of NDUV in this study with the selected alphaviruses used for phylogenetic reconstruction. Click here for file

Additional file 3 **Sequence data. **Fasta format data file showing alignment of amino acids of NDUV in this study with the selected alphaviruses used for phylogenetic reconstruction. Click here for file
