# Supplementary figures and images for: Viral metagenomics demonstrates that domestic pigs are a potential reservoir for Ndumu virus
Source: Virol J. 2012 Sep 24;9:218. doi: 10.1186/1743-422X-9-218 (PMC3512490; doi:10.1186/1743-422X-9-218)

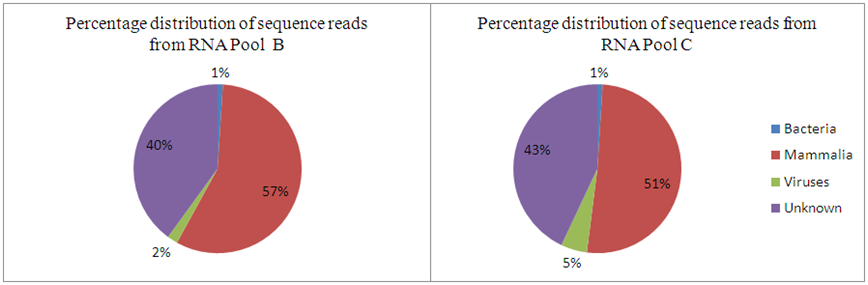

Supplement: Additional file 1 — Figure S1. Taxonomic classification of sequence reads used to build contigs for RNA pools B (Gulu district) and C (Lira district) based on BLASTN (E-value <0.001) against genebank non-redundant database. [file 1743-422X-9-218-S1.tiff]

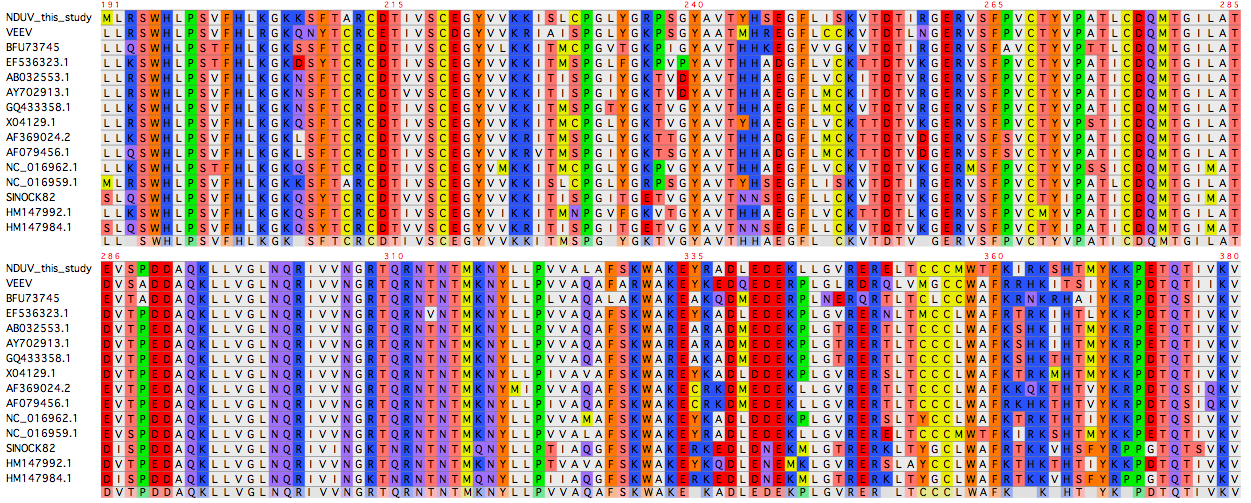

Supplement: Additional file 2 — Figure S2. Window showing alignment of amino acids of NDUV in this study with the selected alphaviruses used for phylogenetic reconstruction. [file 1743-422X-9-218-S2.tiff]
